# Evaluating Ischemia-Modified Albumin as an Early Biomarker for Hypertensive Disorders During Pregnancy: A Case-Control Study

**DOI:** 10.7759/cureus.30867

**Published:** 2022-10-30

**Authors:** Jyoti E John, Apurva Sakarde, Swayam Swaroop Misra, Shuchita Mundle, Jancy Jose, Satyendra Chandra Tripathi, Dnyanesh Amle

**Affiliations:** 1 Biochemistry, All India Institute of Medical Sciences, Nagpur, Nagpur, IND; 2 Obstetrics and Gynaecology, All India Institute of Medical Sciences, Nagpur, Nagpur, IND

**Keywords:** pregnancy, oxidative stress, pre-eclampsia, eclampsia, gestational hypertension, pih, ischemia modified albumin

## Abstract

Background

Ischemia-modified albumin (IMA) is looked upon as a newer marker of myocardial ischemia. There is a paucity of literature however with regard to studies correlating levels of IMA in patients with hypertensive disorders of pregnancy. The present study therefore aimed at estimating the levels of IMA in patients with gestational hypertension and assessing its utility in predicting hypertensive disorders of pregnancy.

Methods

The present study was a hospital-based case-control study conducted in the Department of Biochemistry, All India Institute of Medical Sciences (AIIMS), Nagpur. IMA was estimated in 30 controls (Group I) and 20 cases of gestational hypertension (Group II) using a spectrophotometric assay detecting free unbound Cobalt left behind. The clinical data and lab results were presented as mean ± SD. Student’s t-test was applied and Pearson’s correlation coefficient was calculated. A value of p < 0.05 was taken as statistically significant. The ROC (Receiver Operator Characteristic) curve was used to establish the cut-off of serum IMA levels in pregnancy-induced hypertension (PIH).

Results

There was no significant difference in age and period of gestation (POG) at the time of sample collection between the groups. There was a significant difference in the systolic and diastolic blood pressures (BPs) of both groups. The mean level of serum IMA was significantly higher in cases of gestational hypertension (0.88 ± 0.14 absorbance units {ABSU}) as compared to controls (0.69 ± 0.08 ABSU) (p<0.001). On correlation analysis, the systolic and diastolic BPs were found to be highly positively correlated with serum IMA levels (p<0.001). ROC curve analysis suggested that at a cut-off of 0.73 ABSU, IMA has 85% sensitivity and 80% specificity for predicting gestational hypertension.

Conclusion

Statistically significant results of serum IMA levels obtained in gestational hypertension which falls on the lesser severe spectrum of the disease imply that serum IMA can be used for early diagnosis of gestational hypertension and impending Pre-eclampsia (PE) and Eclampsia.

## Introduction

About 3-10% of all pregnancies in the world are complicated by hypertensive disorders which are a leading cause of maternal morbidity and mortality [1]. Hypertensive disorders of pregnancy include three disorders: Gestational Hypertension, Pre-eclampsia (PE), and Eclampsia.

Various causative mechanisms have been implicated in the etiopathogenesis of hypertensive disorders of pregnancy which include oxidative stress secondary to defective placentation, endothelial dysfunction, intravascular inflammation, and an antiangiogenic state [2]. May be due to the multietiological nature of the disease, there is no single diagnostic test presently available that can predict the likelihood of developing the disease. However, some studies have reported higher levels of ischemia-modified albumin in patients with PE and Eclampsia as compared to normotensive pregnant women [3-5]. Ischemia-modified albumin (IMA) is looked upon as a newer marker of myocardial ischemia [6]. There is a paucity of literature however with regard to studies correlating levels of IMA in patients with gestational hypertension. The objectives of the present study thus were to estimate the levels of serum IMA in patients of gestational hypertension and compare them with those in normotensive pregnant women. Also, to study the correlation of various study variables with serum IMA levels. The aim is to assess whether the serum levels of IMA can be used to predict the likelihood of developing hypertensive disorders during pregnancy. Studies focusing on serum IMA as an early marker of gestational hypertension may help in identifying women who could benefit from early intervention.

## Materials and methods

The present study was a hospital-based case-control study, conducted in the Department of Biochemistry, All India Institute of Medical Sciences (AIIMS), Nagpur, in collaboration with the Department of Obstetrics and Gynaecology and Daga Memorial Government Hospital, Nagpur, between February 2022 to June 2022 in accordance with the Declaration of Helsinki. Permission was sought prior to the study from the Institutional Ethical Committee (Letter no. AIIMS-NAG/IEC/STS/2019/0006 dated 16 May 2019).

Sampling and sample size

The sample size was calculated using the nMaster software, version 2.0 (CMC Vellore, Tamil Nadu, India) based on the mean ± S.D of IMA among PE patients as 106.92 ± 15.02 [4], taking relative precision as 5%, desired confidence level as 95% and non-response rate as 10%. A total of 20 cases (diagnosed cases of gestational hypertension) were enrolled in the study. Thirty normotensive pregnant women were recruited as controls. Sampling was carried out through random selection.

The Controls group (Group I): consisted of normotensive and non-proteinuric healthy pregnant women in the age group of 18-37 years, with a single gestation of >20 weeks, attending the outpatient department (OPD) for regular antenatal care (ANC) check-ups.

The Cases group (Group II): consisted of already diagnosed cases of gestational hypertension in the age group of 18-37 years with a single gestation of >20 weeks attending the OPD or admitted to the wards of the Department of Obstetrics and Gynecology of AIIMS Nagpur and Daga Memorial Government Hospital, Nagpur. The diagnostic criteria followed to diagnose gestational hypertension patients were - pregnant women with blood pressure (BP) ≥140/90 mm of Hg measured on two separate occasions at least four hours apart, or ≥160/110 mm Hg on one occasion at ≥20 weeks of gestation who had normal blood pressure previously [7]. Cases and control groups were matched according to their age and gestational age.

Pregnant women with essential hypertension, multiple pregnancies, bad obstetric history, gestational diabetes mellitus, previous medical history of diabetes mellitus, ischemic heart disease, liver, cardiac or renal diseases, peripheral vascular diseases, or history of smoking were excluded from the study.

Five milliliters of venous blood sample was collected taking all sterile precautions and transferred to labelled vacutainer tubes. The samples were transported to the Clinical Biochemistry lab and centrifuged at 3000 rpm for 10 min. The serum was separated and stored in aliquots at −80°C until analysis. Frozen samples were thawed to room temperature and analysed for Ischemia-Modified Albumin.

Method of estimation of serum ischemia-modified albumin (IMA) (spectrophotometric assay detecting free unbound Cobalt left behind)

Principle: There occurs a peculiar modification in the amino-terminal of human serum albumin during ischemia, such that it then exhibits a decrease in affinity to bind transition metal elements such as Cobalt. This unbound Cobalt is allowed to react with dithiothreitol (DTT), forming a coloured complex that can be measured spectrophotometrically at 470 nm [8].

Assay procedure: The assay method involved adding 50 μL of 0.1% Cobalt chloride to 200 μL of serum. After gently mixing and waiting for 10 minutes, for adequate Cobalt albumin binding to take place, 50 μL of dithiothreitol (DTT) (1.5 mg/mI H_2_O) was added as a colourizing agent. Post two minutes, 1 mL of 0.9% NaCI solution was added to stop the reaction. The serum blank was prepared by adding all the above agents except DTT. The colour development of the test solution was measured spectrophotometrically at 470 nm and reported in absorbance units (ABSU) [8].

Pooled sera with an absorbance close to 0.56 were run in 10 replicates to look for Intra-assay precision. The inter-assay coefficient of variation (CV) was also calculated by running the same pooled sera over six consecutive days. The intra-assay CV was found to be < 3.5%. The inter-assay CV was < 6.0 %.

Statistical analysis

Statistical analysis was carried out using IBM SPSS (Statistical Package for the Social Sciences) software, version 20.0 (IBM Corp., Armonk, NY). The clinical data and lab results were presented as mean and standard deviation. Student’s t-test was used to compare the means of the two groups. Pearson’s correlation analysis was carried out to assess the relationship between two quantitative variables. A value of p <0.05 was taken as statistically significant. The cut-off level for serum IMA in gestational hypertension was established using a ROC curve.

## Results

The demographic data and biochemical characteristics of the two study groups are depicted in Table 1. Table 1 shows that the study groups were found to be matched for age and period of gestation. Both systolic and diastolic blood pressure (BP) were found to be significantly higher in the gestational hypertension group compared to the control group (p<0.001). Serum IMA levels were found to be significantly higher in cases as compared to controls (p<0.001).

**Table 1:** Comparison of demographic and diagnostic parameters in study groups BP: Blood pressure, IMA: Ischemia-Modified Albumin, ABSU: absorbance units

Variables	Group I (Controls) n=30	Group II (Gestational hypertension) n=20	p-value
	Mean ± SD		
Age (years)	24.67 ± 2.87	24.95 ± 3.71	0.762
Period of gestation (POG) (weeks)	29.0 ± 4.76	30.15 ± 3.31	0.353
Systolic BP (mm Hg)	102.47 ± 9.93	146.3 ± 7.57	<0.001
Diastolic BP (mm Hg)	64.67 ± 10.70	92.3 ± 7.18	<0.001
Serum IMA (ABSU)	0.69 ± 0.08	0.88 ± 0.14	<0.001

**Figure 1:**
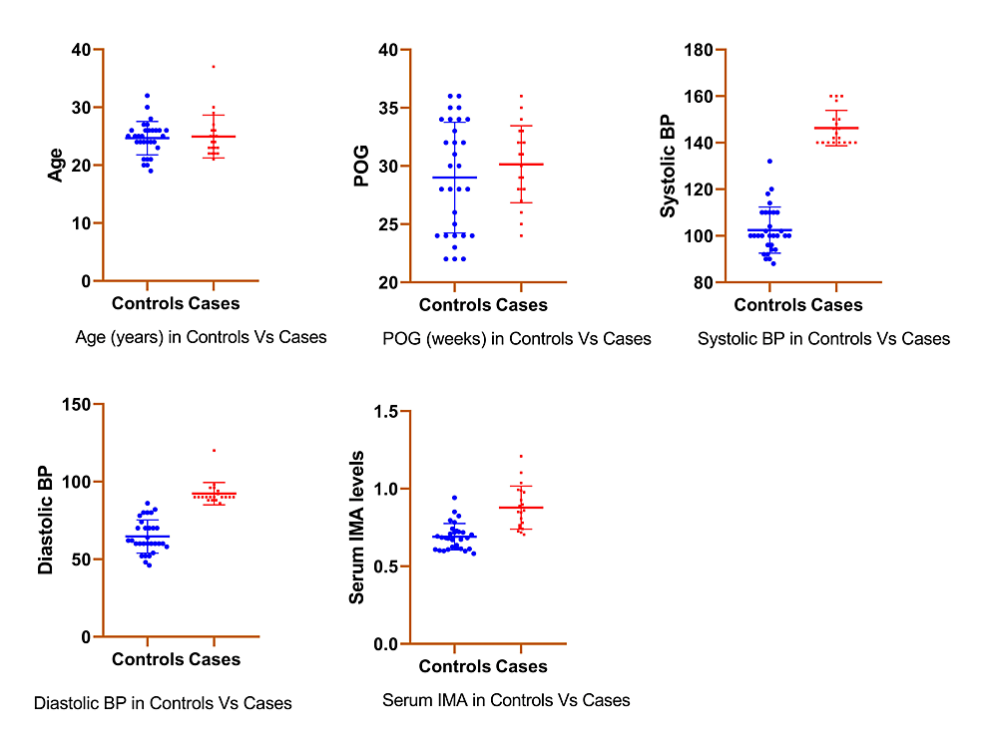
Scatterplot of study variables in both the groups BP: Blood pressure, IMA: Ischemia-Modified Albumin, POG: Period of gestation

Figure 1 is the scatterplot of various study variables in both groups.

Table 2 shows the correlation analysis of IMA with the study variables. IMA was found to have a moderate uphill correlation with systolic BP (r=0.6) and a mild uphill correlation with diastolic BP.

**Table 2:** Correlation analysis of serum IMA with study variables. BP: Blood pressure, IMA: Ischemia-Modified Albumin

Variables	Serum IMA	
	r value	p value
Age (years)	-0.151	0.296
Period of gestation (POG) (weeks)	0.007	0.960
Systolic BP (mm Hg)	0.60	<0.001
Diastolic BP (mm Hg)	0.474	<0.001

**Figure 2:**
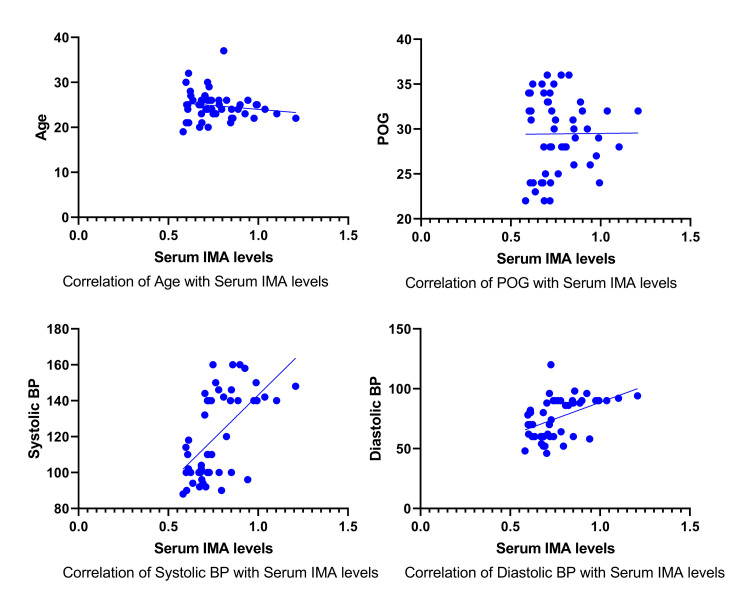
Correlation analysis of serum IMA levels with the various study variables. BP: Blood pressure, IMA: Ischemia-Modified Albumin, POG: Period of gestation

Figure 2 shows the correlation analysis of serum IMA with the various study variables.

**Figure 3:**
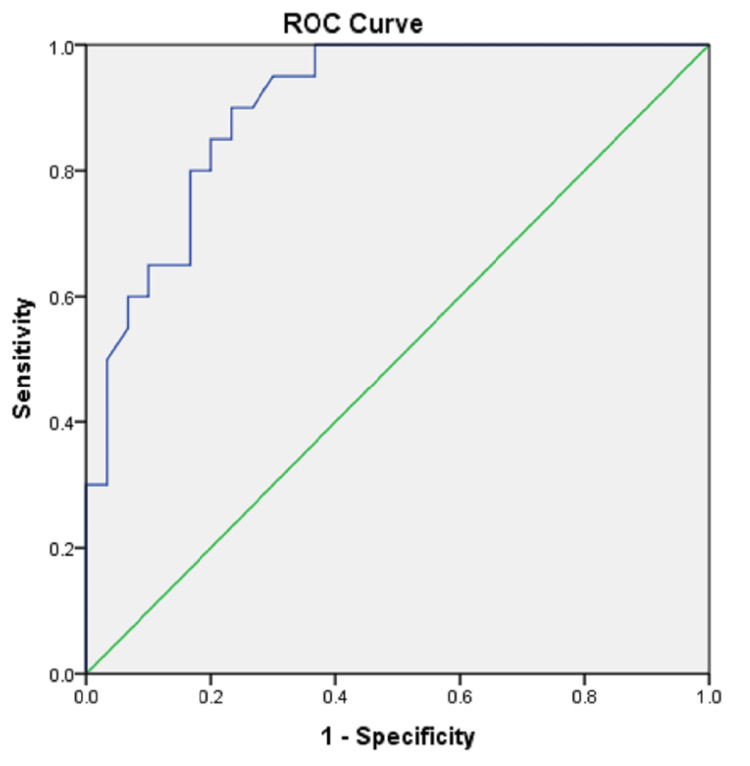
ROC curve of serum IMA ROC: Receiver Operator Characteristic; IMA: Ischemia-Modified Albumin

As depicted in Figure 3, the ROC curve analysis of IMA suggests that at a cut-off of 0.73 ABSU, IMA has 85% sensitivity and 80% specificity for predicting gestational hypertension.

## Discussion

Gestational hypertension affects about 5-8% of all pregnant women in the world [9] and is defined as resting BP ≥ 140/90 mmHg on two occasions at least four hours apart or ≥160/110 mmHg on one occasion in a pregnant woman at ≥20 weeks of gestation who had normal blood pressure previously [10]. About 5-7% of all pregnancies are affected by pre-eclampsia (PE) which is characterised by hypertension and proteinuria [9]. Eclampsia is defined as pre-eclampsia along with the presence of convulsions in the absence of any other neurologic disease [10].

The placental development in normal pregnancy involves the invasion of the spiral arteries by the endovascular trophoblast. A defect in this process and lack of proper remodelling of uterine spiral arteries causes vasoconstriction in the placenta leading to hypoxia and generation of reactive oxygen species (ROS), and thus oxidative stress [11,12]. This damage can be particularly attributed to the high demand for oxygen in the placenta coupled with high lipid concentrations in the presence of defective placentation. This has been confirmed by many studies wherein serum malondialdehyde (MDA) estimated along with other biochemical parameters such as serum lipids in patients of PE and Eclampsia exhibited significantly higher levels, thus suggesting that inappropriate or excessive lipid peroxidation may play an important role in the pathophysiology of hypertensive disorders of pregnancy via oxidative stress [11]. Over the last few decades, changes in lifestyle such as high-calorie diets and delayed childbirth seem to have increased the global incidence of placental-related disorders.

IMA is a novel molecule that has been recently discovered and applied clinically as an early biomarker for ischemia and/or oxidative stress [13,14]. Since gestational hypertension is also a condition associated with ischemia and increased formation of free radicals in the placenta, we hypothesized that there may be an increased production of IMA in these women. Also, the levels of IMA may be expected to rise with the severity of the disease.

The estimation of IMA with the aid of the albumin Cobalt binding approach is primarily based on the fact that human ordinary albumin contains the aspartic acid, alanine, histidine, and lysine (N-Asp-Ala-His-Lys) series of amino acids in its N-terminus vicinity, with an excessive capability to bind to metal ions such as Cobalt (Co), Nickel (Ni), etc. But at some point of ischemia or due to extended oxidative stress, this albumin gets modified due to changes in those binding domains, resulting in a decrease in the affinity to bind Cobalt [15]. This decreased binding capability of the amino (N)- terminal of human serum albumin was studied first by Bar-Or et al. in vitro with the use of exogenous Cobalt in patients with angina and myocardial infarction [8]. By virtue of ischemia bringing about a modification in this albumin, the molecule came to derive its name as Ischemia-Modified Albumin (IMA).

In the present study, serum samples of 30 normotensive and 20 patients with gestational hypertension were analysed for serum IMA. The mean IMA was found out to be 0.69 ABSU in the control group and 0.88 ABSU in the cases. The serum levels of IMA were found to be raised significantly (p<0.001) in patients with gestational hypertension as compared to the controls, suggesting the role of oxidative stress and free radical damage in the cases of hypertensive disorders of pregnancy as compared to controls. In the present study, the ROC curve analysis suggested that IMA has 85% sensitivity and 80% specificity in predicting gestational hypertension at a cut-off value of 0.73 ABSU. The findings of the present study have been found to be consistent with various other similar studies carried out in patients with hypertensive disorders of pregnancies [16-18].

Limitations and strengths

The results of this study can be regarded with due attention to the limitations associated with it, such as a smaller sample size, cross-sectional type of study design, and the study is centered in a particular geographical location. Since the IMA levels were not estimated in patients of different severities of hypertensive disorders of pregnancy, the cause-and-effect relationship could not be established.

Strengths of the present study were age and gestational duration-matched study groups and that although the study population chosen was of lesser severity in the spectrum of the disease, statistically significant results were obtained, thus implying that IMA can be used for early diagnosis of impending PE and Eclampsia. The majority of groups who have studied serum IMA in hypertensive disorders of pregnancy have worked on the PE and Eclampsia groups. However, this study is one of the few studies that has attempted to work on the gestational hypertension group. Further multicentric studies may be carried out to confirm the utility of serum IMA as a predictive biomarker of hypertensive disorders of pregnancy such that it aids in early patient management.

## Conclusions

This study can have profound implications and far-reaching consequences on the management of patients with hypertensive disorders of pregnancy. Many groups have attempted to identify predictive biomarkers for gestational hypertension. However, the majority of these groups have worked on PE and Eclampsia subjects. However, considering the heavy burden of maternal morbidity, mortality, and perinatal and post-natal complications associated with the disease, there was an unaddressed need to pick up the disease in the very early stages.

In this study, the mean levels of serum IMA were significantly higher in the gestational hypertension cases as compared to controls (p<0.001). On ROC curve analysis, at a cut-off 0.73 ABSU, serum IMA was found to have 85% sensitivity and 80% specificity in predicting gestational hypertension which falls on the milder spectrum of the disease. The identification of an early marker of the disease thus will be immensely helpful to pick up the cases for timely intervention. This will in turn aid in preventing co-morbidities associated with the disease in the mother as well as the foetus. Further longitudinal studies with a larger sample size are however required to validate the findings of this study.
